# Loop-mediated isothermal amplification for detection of porcine circovirus type 2

**DOI:** 10.1186/1743-422X-8-497

**Published:** 2011-11-02

**Authors:** Shun Zhou, Si Han, Jianli Shi, Jiaqiang Wu, Xiaoyuan Yuan, Xiaoyan Cong, Shaojian Xu, Xiaoyan Wu, Jun Li, Jinbao Wang

**Affiliations:** 1 Qingdao Agricultural University, Qingdao, 266109, China; 2 Division of Swine Diseases, Shandong Provincial Key Laboratory of Animal Disease Control & Breeding, Institute of Animal Science and Veterinary Medicine Shandong Academy of Agricultural Sciences, Jinan, 250100, China; 3 College of Life Sciences, Key Laboratory of Animal Resistance of Shandong Province, Shandong Normal University, Jinan, 250014, China

**Keywords:** Porcine circovirus type 2 (PCV2), Loop-mediated isothermal amplification (LAMP), Rapid detection

## Abstract

**Background:**

Porcine circovirus type 2 (PCV2) is the primary causative agent of the emerging swine disease known as postweaning multisystemic wasting syndrome (PMWS). Nowadays, polymerase chain reaction (PCR) is still the most widespread technique in pathogen detection. Loop-mediated isothermal amplification (LAMP), a novel nucleic acid amplification method developed in 2000, will possibly replace PCR in the field of detection. To establish a LAMP method for rapid detection of PCV2, two pairs of primers were designed specially from the open reading frame 2 (ORF2) sequences of PCV2. A LAMP method for rapid detection of PCV2 was established. To compare with PCR, sensitivity and specificity of LAMP were evaluated using the optimized reaction system. The LAMP products could be determined by agarose gel electrophoresis or adding SYBR Green I dye.

**Results:**

The amplification of LAMP could be obtained at 63°C for 60 min. The detection limit was nearly 1 copy of DNA plasmid, more sensitive than PCR. There was no cross-reaction with porcine circovirus type 1 (PCV1), porcine pseudorabies virus (PRV) and porcine parvovirus (PPV) under the same conditions.

**Conclusions:**

LAMP is an useful rapid detection method with high sensitivity and specificity for PCV2.

## Background

Porcine circovirus (PCV) is a non-enveloped, single-stranded circular DNA virus, which is divided into two distinct genotypes according to pathogenicity, antigenicity and DNA sequence [1]. PCV1 was considered non-pathogenic. But PCV2 is considered as the major pathogen of postweaning multisystemic wasting syndrome (PMWS) [2]. In addition, PCV2 was also associated with porcine respiratory disease complex (PRDC), porcine dermatitis and nephropathy syndrome (PDNS), proliferative and necrotizing pneumonia (PNP), etc [3]. Piglets infected with PCV2 were immunosuppressive beacause of virus invading immune system [4]. It makes other bacteria and viruses have chance to infect hosts. Epidemiological investigation showed PCV2 was fairly common in swine herds recent years. Pig industry in China suffered economic losses owing to diseases caused by PCV2 [5]. At present, the detection technique used widely in the laboratory is conventional PCR and quantitative real time polymerase chain reaction (Q-PCR). However, these methods have some shortcomings, such as costly PCR instruments and a long detection period. These limitations affect their applications to certain environments.

Loop-mediated isothermal amplification (LAMP) is a novel nucleic acid amplification method that was developed originally by Notomi et al in 2000 [6]. The LAMP assay has qualities of high sensitivity and specificity, moreover, it's a quite rapid detection technique. The method employs four primers specific to six regions and *Bst *DNA polymerase which has a function of strand displacement. The whole amplification could be finished within an hour under isothermal conditions about 60-65°C. There are a few ways to determine the result. An important merit of LAMP is cost-effectiveness, which gets rid of expensive instruments and equipments. Nowadays, LAMP has already been regarded as a good detection approach applying to many kinds of pathogens [7-9]. In this study, we developed a LAMP assay for rapid detection of PCV2, based on the original LAMP [6].

## Results

### The optimized LAMP assay

**Figure 1:**
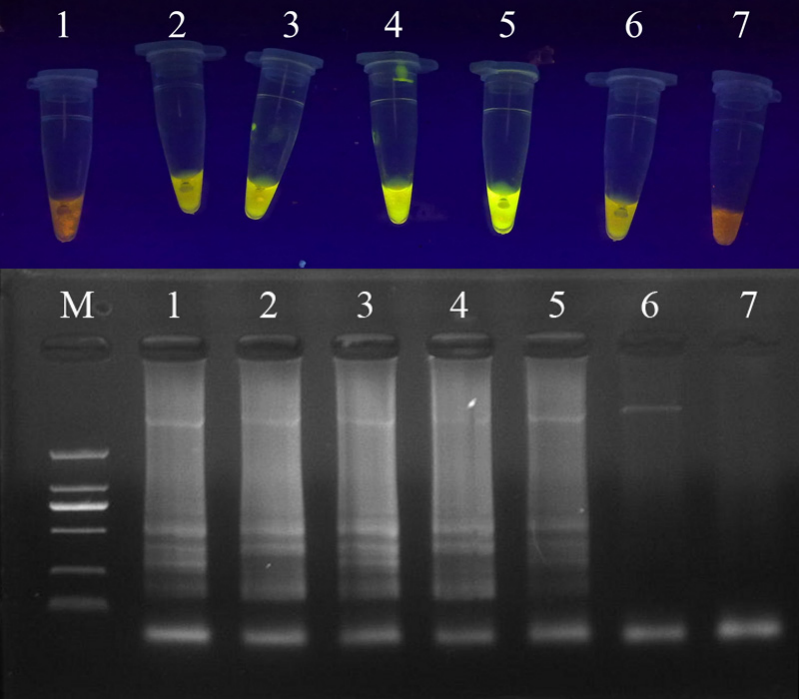
**The optimum reaction temperature of LAMP for PCV2 by electrophoretic analysis and visual inspection**. Lane M, DNA Marker DL-2000 (Takara); Lanes 1-6, different reaction temperature of LAMP (60°C, 61°C, 62°C, 63°C, 64°C, 65°C); Lane 7, negative control.

The optimum LAMP reaction mixture (25 μL) contained 2.4 μM (each) of inner primers FIP and BIP, 0.24 μM (each) of outer primers F3 and B3, 0.6 mM each deoxynucleoside triphosphate, 0.4 M betaine, 1 × ThermoPol buffer (20 mM Tris-HCl, 10 mM KCl, 10 mM (NH_4_)_2_SO_4_, 2 mM MgSO_4_, 0.1% Triton X-100), 8 U of *Bst *DNA polymerase (New England Biolabs, USA) and 1 μL of template DNA. The mixture was incubated at 63°C for 60 min, and then heated at 80°C for 5 min to terminate amplification. The result of agarose gel electrophoresis showed that a characteristic ladder-like pattern of bands was clearest when the reaction was performed at 63°C (Figure 1) [6]. The same result was appeared by adding SYBR Green I dye.

### Sensitivity of LAMP and PCR for PCV2

**Figure 2:**
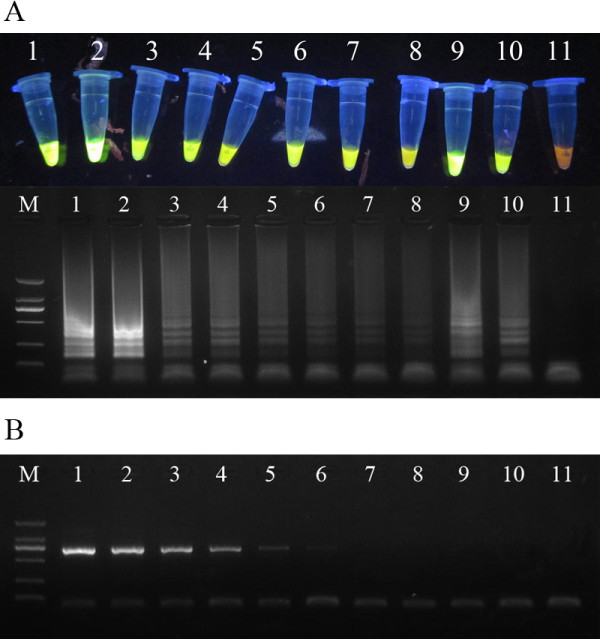
**Comparative sensitivity of LAMP and PCR for PCV2**. (A) Electrophoretic analysis and visual inspection of LAMP amplified products. (B) Electrophoretic analysis of PCR amplified products. Lanes: M, DNA Marker DL-2000 (Takara); 1, 1 × 10^9 ^copies/tube; 2, 1 × 10^8 ^copies/tube; 3, 1 × 10^7 ^copies/tube; 4, 1 × 10^6 ^copies/tube; 5, 1 × 10^5 ^copies/tube; 6, 1 × 10^4 ^copies/tube; 7, 1 × 10^3 ^copies/tube; 8, 1 × 10^2 ^copies/tube; 9, 1 × 10^1 ^copies/tube; 10, 1 × 10^0 ^copy/tube; 11, negative control.

The target gene of PCR was the complete PCV2-ORF2 sequences. Using serial dilutions of 1 × 10^0 ^to 1 × 10^9 ^copies of DNA from recombinant plasmid as templates, the LAMP assay and conventional PCR for PCV2 were carried out for compare of detection limits. All amplified products were analyzed by agarose gel electrophoresis. The detection limit of LAMP was 1 × 10^0 ^copy, and the concentration of plasmid was about 0.01 fg/μL. On the other hand, the conventional PCR had a detection limit of 1 × 10^4 ^copies, and the the concentration of plasmid was nearly 100 fg/uL (Figure 2). So the LAMP assay for PCV2 was 10000 times as sensitive as the conventional PCR.

In 58 clinical known samples, LAMP detected 18 positive samples, whereas PCR detected 12 positive samples. There were 6 samples that only LAMP could detect well.

### Specificity of LAMP for PCV2

**Figure 3:**
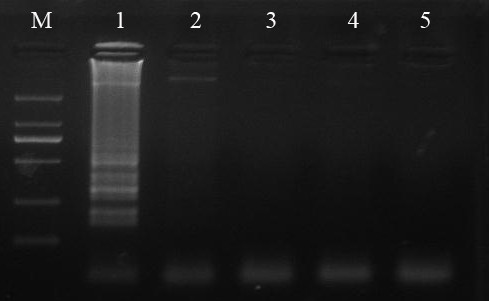
**Specificity of LAMP for PCV2 by electrophoretic analysis**. Lanes: M, DNA Marker DL-2000 (Takara); 1, plasmid DNA of PCV2; 2, plasmid DNA of PCV1; 3, DNA of PRV; 4, DNA of PPV; 5, negative control.

PCV, PRV and PPV were some common DNA viruses in causing swine diseases. In this assay, plasmid pSK-PCV2 SD1 was used as DNA template of PCV2, and plasmid pMD18-T-PCV1 was used as DNA template of PCV1. The result of agarose gel electrophoresis indicated only PCV2 gave a positive reaction, a ladder-like pattern of bands. And yet under the same conditions, PCV1 didn't give a positive reaction with the primers of FIP, BIP, F3 and B3. Neither PRV nor PPV did (Figure 3). The assay demonstrated LAMP for PCV2 did well in specificity. Four primers need to recognize six distinct regions in LAMP reaction, so it amplifies target DNA with high specificity.

## Discussion

There are two distinct clusters in porcine circovirus: type 1 and type 2. Pathogenic PCV2 is very harmful to pig industry. Contrast PCV1 and PCV2, nucleotide sequence homology is no more than 80% [10]. PCV2 has two major open reading frames (ORF1 and ORF2) [11,12]. Between PCV1 and PCV2, nucleotide sequence homology is about 83% in ORF1 region, while it is about 67% in ORF2 region [10]. In order to only amplify DNA of PCV2, all primers in the experiment were designed from the ORF2 sequences. They worked as we had expected.

Principle of LAMP demonstrates that forming an initial stem-loop structure later, inner primers hybridize to regions of target sequences at the same time [13]. The final products are DNAs with different numbers of stem-loop structure. A characteristic ladder-like pattern of bands can be seen through agarose gel electrophoresis. Besides electrophoresis, adding SYBR Green I is another good way to determine the result. It can be observed directly with naked eyes. While it is more obvious that amplified products in tubes send out fluorescence under UV light. Enosawa et al. [14] had described that magnesium pyrophosphate, produced during the amplification of LAMP, could form visual white precipitates after centrifugation. However, in our study we found that it was pretty hard to observe the white precipitates by naked eyes.

The final concentrations of ingredients in LAMP assay had something to do with the primers. Another set of primers may not hybridize to the template under the same concentrations of reaction mixture. In this experiment, when reaction mixture contained more than 2 mM MgSO_4_, the amplification of LAMP reaction wouldn't occur. Similar situation still happened to the concentration of primers. When reaction mixture contained less than 2.0 μM (each) of inner primers FIP and BIP, 0.2 μM or less (each) of outer primers F3 and B3, the amplification would be obtained only when template DNA was in some comparatively large concentration. So it is important to optimize LAMP reaction system before assessment of sensitivity and specificity.

LAMP, as a novel nucleic acid amplification method, has been applied to detections of many kinds of pathogens in recent years. Compared with widely used PCR, LAMP amplifies more rapidly only under isothermal conditions. If additional two loop primers are used in LAMP reaction, the detection time will be shortened to less than 30 min [15]. LAMP is more sensitive than PCR. Moreover, the detection instruments are quite common in laboratory, just a water bath and UV light. We noticed that sometimes reaction tubes could affect mutually and resulting in false positive determination. Maybe it has much to do with ascending vapor by heat. In order to avoid this circumstance, the reaction tubes should be sealed with sealing compound before heated. Meanwhile, cap of tube should keep a certain distance from float.

## Conclusions

In summary, LAMP is a very simple and efficient detection method with high sensitivity and specificity for the clinical diagnosis of PCV2, that will suit the requirements of rapid detection in laboratory and general conditions.

## Materials and methods

### Plasmid and clinical samples

A recombinant plasmid pSK-PCV2 SD1 containing complete sequence of PCV2 was constructed in our laboratory. Another recombinant plasmid pMD18-T-PCV1 that contained complete sequence of PCV1 was constructed in our laboratory, too. PPV and PRV were identified by conventional PCR and sequencing.

A total of 58 clinical known samples from different areas of Shandong in China were used for diagnostic purpose, which 20 samples were positive for PCV2.

### Plasmid extraction

Following the manufacturer's instruction, the plasmid was extracted by using a TIANprep mini plasmid kit (TIANGEN Biotech Co.LTD., China). The purified plasmid was eluted in 60 μL of sterile water and stored at -20°C until later use.

### DNA extraction

First, 500 μL of tissue samples in an Eppendorf tube that have been frozen and thawed three times was digested by 50 μL of 10% SDS and 10 μL of proteinase K (20 mg/mL) at 55°C for 2 h. Then, DNA was extracted with TRIS saturated phenol, chloroform and absolute ethanol according to priority. At last, DNA was washed with 75% ice-cold ethanol. The precipitation of DNA was dissolved in 20 μL of sterile water and stored at -20°C for later use.

### Primer design

On the basis of the published PCV2 sequences, two pairs of primers for LAMP were designed with online software, PrimerExplorer V4 (http://primerexplorer.jp/e/). The primers targeted the ORF2 gene sequences of JS2003 that was obtained from GenBank (accession number AY578327), including a pair of inner primers (FIP and BIP) and a pair of outer primers (F3 and B3). Another two primers (P3 and P4) were forward and reverse primers respectively for conventional PCR. All primers sequences are listed in Table 1.

**Table 1:** Details of LAMP and PCR primers

Primer name^a^	Genome position^b^	Sequence (5'→3')
F3	1355-1378	CTCCAGTGCTGTTATTCTAGATGA
B3	1160-1181	TTTCCAGCAGTTTGTAGTCTCA
FIP	1273-1295 1317-1335	GAAGGGCTGGGTTATGGTATGGC-TTTT^c^- ACAGCCCTCACCTATGACC
BIP	1240-1261 1182-1204	CCGCTACTTTACCCCCAAACCT-TTTT^c^- GCCACAGCTGATTTCTTTTGTTG
P3	1721-1742	GCGGATCCGTGACGTATCCAAG
P4	1025-1049	CCACTAGTATAGGGGTTAAGTGGGG

^a ^F3, forward outer primer; B3, backward outer primer; FIP, forward inner primer; BIP, backward inner primer. ^b ^Genome position according to the JS2003 complete genome sequence (GenBank accession number AY578327). ^c ^Both inner primers of FIP and BIP containing 2 binding regions and connected by a TTTT bridge

### LAMP reaction and optimization

LAMP was carried out in a water bath. Referring to the basic reaction system for LAMP, the concentrations of following ingredients were optimized in a 25 μL total reaction volume: primers, deoxynucleoside triphosphate, MgSO_4 _and betaine. The mixture was performed at 59-65°C for 60 min and then heated at 80°C for 5 min to terminate the reaction [16-19].

The amplified products were electrophoresed in 1.5% Tris-Borate-EDTA (TBE) agarose gel and the gels were stained with ethidium bromide solution. Moreover, adding 1 μL of SYBR Green I dye to the LAMP products, the amplification was directly detected with naked eyes [7]. To confirm the result, the reaction tubes should be observed under UV light.

### PCR

To compare the sensitivity and specificity between LAMP and PCR, PCR was carried out in a 25 μL reaction volume containing final concentrations of 0.4 μM each primer, 0.2 mM each deoxynucleoside triphosphate, 1.5 mM MgCl_2_, 1× PCR buffer, 1 μL of extracted DNA and 2.5 U of *Taq *DNA polymerase (Fermentas, Canada). The amplification was achieved by 30 cycles at 95°C for 3 min, 94°C for 1 min, 55°C for 1 min, 72°C for 1 min, with a final elongation for 10 min at 72°C. The PCR products were analyzed by 1.5% agarose gel electrophoresis.

### Sensitivity and specificity of LAMP

To calculate the plasmid copy number in 1 μL of solution, the concentration of plasmid pSK-PCV2 SD1 was determined by using Nanodrop 2000 spectrophotometer (Thermo Scientific, USA). Serial 10-fold dilutions containing copies of 1 × 10^0 ^to 1 × 10^9 ^were used as templates in the assay [17]. To compare the sensitivity of LAMP with PCR, Both LAMP and PCR were carried out under optimized conditions. Furthermore, 58 clinical known samples were detected by LAMP and PCR simultaneously.

DNA of PCV1, PRV, PPV may cause potential cross-reactions in LAMP assay for PCV2. DNA of these viruses were examined to assess the specificity of LAMP. Plasmid pSK-PCV2 SD1 was used as the positive control while sterile water was used as the negative control.

## List of abbreviations

BIP: backward inner primer; FIP: forward inner primer; LAMP: Loop-mediated isothermal amplification; ORF2: open reading frame 2; PCR: polymerase chain reaction; PCV: porcine circovirus; PCV1: porcine circovirus type 1; PCV2: porcine circovirus type 2; PDNS: porcine dermatitis and nephropathy syndrome; PMWS: postweaning multisystemic wasting syndrome; PNP: proliferative and necrotizing pneumonia; PPV: porcine parvovirus; PRDC: porcine respiratory disease complex; PRV: porcine pseudorabies virus; Q-PCR: quantitative real time polymerase chain reaction.

## Competing interests

The authors declare that they have no competing interests.

## Authors' contributions

SZ, SH, JS, JW, XC, SX, XY, XW carried out the experiments and wrote the manuscript. JL, JW conceived the studies and participated in experimental design and coordination. All authors read and approved the final manuscript.
